# On the redundancy of denoising in electrocardiogram reconstruction

**DOI:** 10.3389/fcvm.2026.1827352

**Published:** 2026-06-26

**Authors:** Ekenedirichukwu N. Obianom, Abdulhamed M. Jasim, Abubakar Sadiq Muhammad, G. André Ng, Xin Li

**Affiliations:** 1 Division of Cardiovascular Sciences, University of Leicester, Leicester, United Kingdom; 2 School of Engineering, University of Leicester, Leicester, United Kingdom; 3 Department of Cardiology, University Hospitals of Leicester NHS Trust, Leicester, United Kingdom; 4 Leicester British Heart Foundation Centre of Research Excellence, Leicester, United Kingdom; 5 Leicester National Institute for Health and Care Research Biomedical Research Centre, Leicester, United Kingdom

**Keywords:** correlation, denoising, ECG, LSTM, machine learning, reconstruction, transfer learning

## Abstract

**Introduction:**

Electrocardiogram (ECG) reconstruction involves synthesizing specific leads from other recorded leads, addressing cases where standard ECGs have missing, noisy, or unrecorded leads. Traditionally, reconstruction pipelines include a denoising step, which could be time-consuming and may distort the original signal morphology. Recent studies have shown that reconstruction can be successfully performed without denoising by using multiple models tailored to different input noise levels. This paper builds on that approach by evaluating whether a single, unified model can achieve similar performance.

**Methods:**

The study compares the multiple-model pipeline (MMP) and the single-model pipeline (SMP) using 10,000 normal ECG records from the CODE-15% database (trimmed to 10 s, resampled to 500 Hz, and denoised to act as a reference point for when artificial noise is added prior to reconstruction).

**Results:**

Results show that the SMP performs comparably to the MMP, achieving average correlations above 0.85, consistent with previous research, and maintaining stable performance across noise levels (∼0.04 variation). Although the model performs poorly when tested on abnormal ECG, fine-tuning the model through transfer learning with as little as ten seconds data returns its performance to the expected level by making the generic model patient specific.

**Discussion:**

These findings have significant implications for wearable medical devices, where minimizing storage and computational demands is critical. While the MMP requires over five times the memory due to multiple models, the SMP (and transfer learning) offers a more streamlined, efficient, and cost-effective solution by eliminating both the need for multiple models and separate denoising steps.

## 1 Introduction

It is estimated that around 300 million electrocardiogram (ECG) tests are performed globally yearly (13). This is because ECG has become an essential tool for cardiac diagnosis as it is a non-invasive and painless method of measuring the electrical activity of the heart (9). However, the standard ECG (which is made up of 10 electrodes which mathematically form 12 leads) can often have missing, noisy or unrecorded leads, making it necessary for the reconstruction of these leads. Reconstruction of ECG is the process of synthesizing specific ECG leads from other recorded ECG leads. This synthesis can help retrieve the missing leads which inadvertently supports diagnosis for speedy patient care.

Over the years, a wide range of electrocardiogram (ECG) reconstruction techniques have been explored to recover missing or unmeasured ECG leads from a reduced set of input leads (4, 8, 16, 18, 19). These approaches range from linear methods, such as transformation matrices and Independent Component Analysis, to nonlinear approaches including Support Vector Machines and neural networks. Despite the diversity of reconstruction architectures, previous studies have demonstrated that model design alone does not fully determine reconstruction performance (Table 1). Obianom et al. (24) and Feild et al. (10) showed that reconstruction models can maintain reliable performance in the presence of arrhythmic signals, although performance is often reduced compared to reconstruction under regular rhythmic conditions. This suggests that signal morphology and rhythm irregularity introduce additional challenges that influence reconstruction fidelity independently of the underlying reconstruction architecture.

**Table 1:** Comparative summary of ECG reconstruction literature, highlighting algorithmic approaches, input lead configurations, noise handling strategies, evaluated cardiac conditions, model training paradigms, reported reconstruction performance, and datasets used.

Study	Algorithm	Input leads	Noise handling strategy	Pathology evaluated	Model type	Reported correlation	Dataset
Nelwan et al. (19)	Linear regression	I, II, V2, V5	Cascaded filtering pipeline: Moving average filter, and linear interpolation method	Myocardial Infarction (MI)	Generic (Gen) and Personalised (PS)	Median = 0.959 (Gen), 0.985 (PS)	REPAIR (5)
Atoui et al. (4)	Feed Forward Network	I, II, V2	Cascaded filtering pipeline (3)	Normal and Acute Coronary Syndrome	Generic and Personalised	Mean = 0.932 (Gen), 0.962 (PS)	Personal
Maheshwari et al. (16)	Linear Transformation	I, II, V2	Not explicitly reported	Myocardial Infarction, Coronary Artery Disease, Ventricular Tachycardia, Myocardial Ischemia, and Normal	Personalised	Mean = 0.982	INCART (11) and PTBDB (6, 11)
Lee et al. (15)	Linear Regression and State space model	I, II, III	Band-pass filter (0.5–150 Hz)	Multiple cardiac conditions	Personalised	Mean = 0.992	PTBDB (6, 11)
Vemishetty et al. (32)	Linear Transformation	I, V2	Cascaded filtering pipeline: Band-pass filter (0.05–40 Hz) and median filter	Normal (Norm), Bundle Branch Block (BBB), and Myocardial Infarction	Personalised	Mean = 0.957 (Norm), 0.920 (BBB), 0.889 (MI)	PTBDB (6, 11)
Dhahri et al. (8)	Long Short-Term Memory	I, II, V2	Not explicitly reported	Not explicitly reported	Generic	Mean = 0.950	PTBDB (6, 11)
Obianom et al. (25)	Linear Transformation with wave masking	I, II, V3	Cascaded filtering pipeline:	Normal ECG	Generic	Mean = 0.88 Median = 0.945	CODE-15% (29)
Present study	Long Short-Term Memory	I, II, V3	No prior denoising	Normal and Myocardial Infarction	Generic and Personalised	Mean = 0.871 (Gen- Norm), 0.937 (PS-Norm), 0.590 (Gen-MI), 0.823 (PS-MI)	PTB-XL (11, 33) and CODE-15% (29)

This can be observed in the work of Vemishetty et al. (32) and Lee et al. (15), who demonstrated that reconstruction performance varies across different ECG rhythms and cardiac conditions (Table 1). Their approaches involved training reconstruction models on a diverse range of heart conditions, supporting the premise that models exposed to greater morphological variability during training are more likely to generalise effectively to abnormal or arrhythmic ECG signals. This is further reflected in the poorer performance achieved by the generic model reported in Obianom et al. (23), where limited pathological representation reduced reconstruction robustness under abnormal conditions. Nevertheless, rhythm pattern and cardiac morphology are not the only signal characteristics influencing reconstruction performance. ECG signals are frequently contaminated by acquisition noise, and inconsistent reconstruction can occur when noisy input signals are not adequately accounted for during model development.

Traditionally, reconstruction pipelines apply cascaded filtering stages to remove baseline wander, power line interference, electromyographic interference, and Gaussian noise prior to model inference. This preprocessing step standardises the input signal by reducing acquisition variability, ensuring that reconstruction models operate on a more consistent and constrained signal representation. While this can improve training stability and prediction reliability, it also introduces additional computational overhead and delays depending on the denoising method adopted: a 1-second ECG segment may take as little as 6.62 ± 0.59 ms to denoise using rule-based methods or up to 204.03 ± 18.39 ms with a DenseNet model (28, 36). More recent work by Obianom et al. (22) challenged the necessity of this preprocessing stage by demonstrating that deep learning models trained directly on noisy ECG signals can still learn to reconstruct reliable ECG leads without prior denoising. This approach reframes denoising as an implicit learning problem rather than an explicit preprocessing requirement, thereby reducing pipeline complexity while maintaining reconstruction performance.

Their research focused on reconstructing ECG without prior denoising by adding specific levels of Gaussian noise (0 dB to 20 dB) to input signals and training models to handle each noise level. This approach was tested on both healthy and MI patients. Both linear algorithms and DL models delivered promising performance, but DL models showed greater consistency, with only ∼0.05 variation in correlation across noise levels. Average output correlations exceeded 0.85, aligning with results from peer studies (4, 8, 16, 18, 19). By eliminating the denoising step, this method reduces reconstruction time but increases the number of models needed for a generalized system, which poses storage challenges, especially for embedded systems. To address this, the number of models need to be reduced by increasing the range of input noise a model can handle. This raises a critical new question: *What is the maximum range of input noise that a single reconstruction model can reliably and effectively manage?*

**Figure 1:**
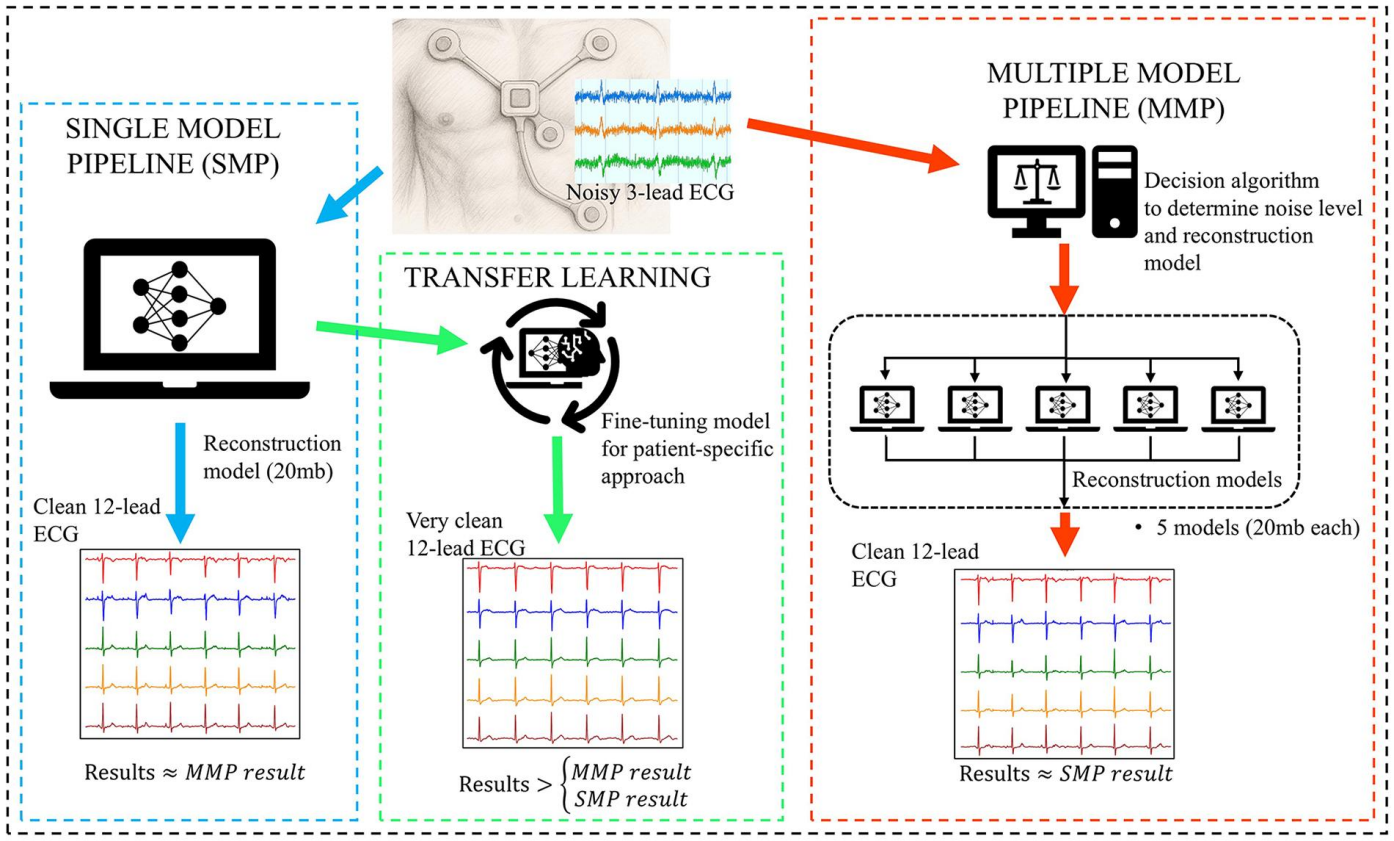
Overview of the work done in this paper. Left (Blue): Single model pipeline (SMP) which takes in ECG with various noise levels and outputs denoised synthesised leads. Middle (Green): Transfer learning continuation of the SMP. It fine-tunes the generic model of the SMP to a patient-specific form to improve the performance of the model on each patient irrespective of heart condition. Right (Red): Multiple model pipeline which classifies the noise level of the ECG before transferring it to the model designed for that noise level for synthesising denoised leads.

This paper improves on the work of Obianom et al. (22) by exploring the extent to which a single model can effectively reconstruct ECG signals across a broad spectrum of input noise levels. The study aims to determine whether a unified model can maintain high reconstruction accuracy without the need for multiple noise-specific models. If successful, such an approach would offer several practical advantages: simplification of system architecture, reduction of storage and computational requirements, and elimination of the need for pre-assessment or classification of input noise levels before reconstruction. The work here tests this hypothesis by training a deep learning model on a dataset containing ECG signals with a diverse range of added Gaussian noise and evaluates the model's performance in reconstructing clean ECG leads across all noise levels, comparing its results to those of noise-specific models. Ultimately, the goal is to determine whether a single-model solution can match or approach the performance of a multi-model setup, making it a more efficient and scalable option for real-world applications, including embedded systems and wearable health technologies. Additionally, transfer learning is integrated to investigate how the generic form of this model can be extended to patients with diverse heart conditions (Figure 1).

## 2 Materials and methods

### 2.1 Dataset

#### 2.1.1 Generic model data

The dataset employed in this study comprises 10,000 patients, randomly selected from the CODE-15% dataset (29), based on specific inclusion criteria outlined in the accompanying metadata. The selection process ensured the following:

(a) Each ECG must originate from a unique patient not previously selected from the dataset.
(b) The ECG must be classified as normal.
(c) The patient must not exhibit any of the monitored conditions at the time of data collection, including first-degree AV block, right bundle branch block, left bundle branch block, sinus bradycardia, atrial fibrillation, or sinus tachycardia.
(d) All leads within the ECG must contain valid, non-blank signals.

This large dataset was chosen because the larger the amount of testing data, the more representative the results are of real-world performance. A larger dataset captures a wider variety of signal patterns and individual differences across patients. This dataset will be called SET1.

#### 2.1.2 Transfer learning analysis data

After model analysis, further testing was performed on the final model to evaluate its performance on a different dataset. The dataset used for this evaluation is extracted from PTB-XL database (11, 33). PTB-XL was collected over the course of seven years from 1989 to 1997. It consists of patients with diverse heart conditions that may or may not affect the condition of the heart. The extracted dataset was chosen with the following criteria to ensure randomness and increase the generalisation of the outcome:

(a) The first ECG record of each ECG set must not belong to a patient that had been previously selected.
(b) Each ECG set must belong to a single patient.
(c) The ECG must be categorised as normal or Myocardial Infarction (MI).
(d) The patient must be false for every other ailment.
(e) No lead of the ECG must have a blank signal.

The outcome of this was 10 patients where the initial and final ECG records were classified normal, and 10 patients where the initial and final records were classified MI. MI was selected as the pathological condition for evaluation because, although it introduces clinically significant morphological abnormalities into the ECG waveform, the underlying rhythm often remains relatively regular. This provides a controlled pathological scenario in which the reconstruction models are challenged with altered waveform characteristics without the additional complexity introduced by highly irregular rhythms. Consequently, MI serves as an intermediate test condition that incrementally extends the difficulty of the reconstruction task while still allowing meaningful assessment of model robustness and adaptability. The time difference between initial and final record ranges between seconds and a day. In total, the outcome of the choosing criteria was 20 patients with 2 records each. This dataset will be called SET2.

#### 2.1.3 Data preprocessing

Following selection, the ECG signals were standardized to a duration of 10 s, resampled to 500 Hz, and subsequently denoised using the ECG deli MATLAB toolbox (26) at high pass filter of 0.3 Hz, low pass filter of 120 Hz and notch filter of 60 Hz for SET1 and 50 Hz for SET2 because of the power line frequency in the country of origin. The data was denoised to have a common baseline for analysis across all records prior to noise addition and model training and testing. Importantly, this method of denoising was chosen because it primarily removes baseline wander, narrowband power line interference and higher frequency electromyographic noise, which are considered acquisition artefacts, while aiming to preserve the primary clinically relevant morphological components of the ECG waveform. As such, the intent is not to reshape or enhance the waveform, but to standardise the input space. Given the absence of a universally accepted optimal denoising pipeline for ECG, this approach represents a widely adopted (as seen in Table 1) and computationally efficient compromise that ensures reproducibility across large datasets. With over 10,000 signals, manual or clinician-certified denoising would be impractical, requiring extensive expert time and potentially introducing inter-observer variability. Therefore, this automated filtering step ensures scalability and consistency while maintaining preservation of key waveform morphology.

With respect to lead choice, Schreck et al. (31) demonstrated that using just three leads as input for ECG reconstruction was sufficient to capture most of the information content in the output, achieving 99.12 ± 0.92% accuracy. Although the inclusion of additional leads provided slight improvements, the overall benefit was minimal. Given that the limb leads (III, aVF, aVL, and aVR) can be mathematically derived from leads I and II, reconstruction efforts have traditionally concentrated on the precordial leads. Historically, several studies (including those by Scherer et al. (30) and Nelwan et al. (20)) have employed leads I, II, and V2 as inputs to reconstruct the remaining precordial leads (V1, V3, V4, V5, and V6). This combination has been widely adopted due to its demonstrated effectiveness, often without further exploration of alternative lead configurations. However, Butchy et al. (7), supported by Mason et al. (18) and Obianom et al. (21), revisited this approach through a lead-to-lead correlation and mean-squared-error analysis and found that lead V3 exhibited stronger performance with the other leads compared to V2. Building on this insight, the present study adopts this proposed configuration, using leads I, II, and V3 as input for ECG reconstruction, to improve reconstruction accuracy based on inter-lead correlation strength.

### 2.2 Pipeline design

**Figure 2:**
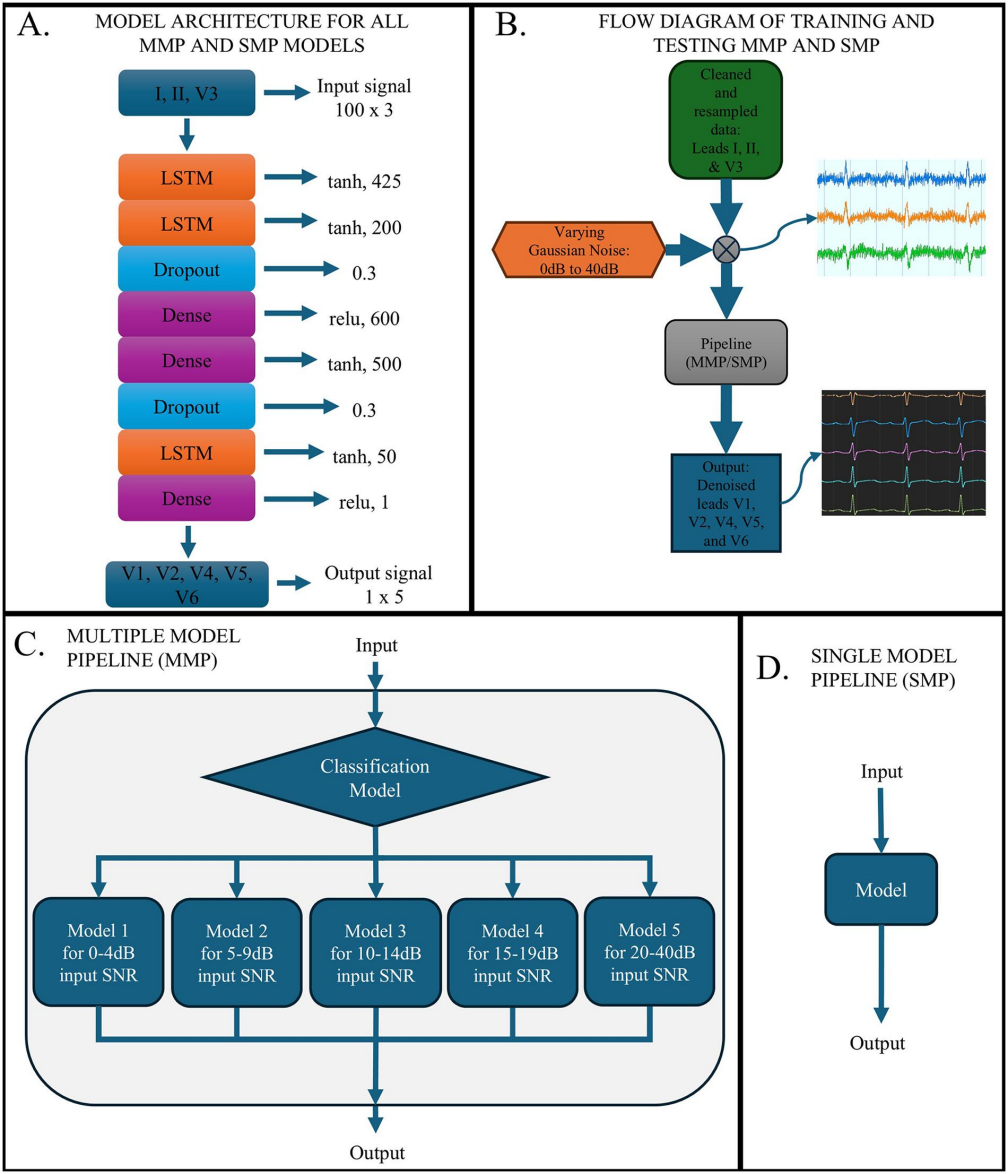
**(A)** Model architecture used in the pipelines. Each block shows the type of layer while the arrow to the right shows the activation function and number of neurons. **(B)** Flow diagram for training and testing the pipelines, showing how various noise levels are added to the denoised/cleaned ECG signal prior to inputting it to the model for either model training or model testing. **(C)** Multiple Model Pipeline (MMP) showing how a classification algorithm is required to decide the noise level before passing it on to the relevant model (Model 1 to Model 5) for reconstruction. **(D)** Single Model Pipeline (SMP) in general use showing the input signal directly applied to the model irrespective of noise level.

The models used in the different pipelines in this study have identical architecture, based on a long short-term memory (LSTM) network. LSTM is an advanced form of recurrent neural network designed to retain historical information from sequential inputs, enabling it to make decisions informed by both past and present data. The LSTM architecture employed for ECG reconstruction in this study is a modified version of the model proposed by Obianom et al. (22), as illustrated in Figure 2A, which itself was adapted from Dhahri et al. (8) who showed that an average reconstruction results of 0.95 correlation can be achieved with it. Specifically, an additional LSTM layer and a fully connected layer were incorporated to strengthen the network's temporal feature extraction capacity and improve its ability to capture longer-range dependencies within ECG signals. The added LSTM layer increases representational depth across sequential patterns, while the dense layer improves nonlinear mapping from learned temporal features to the reconstructed ECG outputs. Beyond these structural modifications, the model hyperparameters, including training configuration and sequence processing settings (Figure 2A), were systematically tuned to better align with the substantially larger dataset and the reconstruction objectives of this study. These adjustments include optimisation of learning dynamics and sequence handling to improve stability under higher data variability. Collectively, these enhancements were designed as targeted architectural refinements aiming to improve generalisation across diverse signal conditions while preserving the stability and reconstruction fidelity demonstrated by the original baseline architecture.

In this improved architecture, 100 samples of each input were simultaneously used to predict all output leads at once. To preserve the temporal structure of the data, dense layers are implemented using a time-distributed approach. Given the scale of the dataset (which comprises 10,000 ten-second, 500 Hz, 12-lead ECG recordings), training for many epochs would require weeks of computation based on the available resources and is not practical. To balance computational cost with model performance, a distributed hyperparameter search was conducted on the version of SET1 containing 0 dB additive white Gaussian noise (WGN) to identify optimal hyperparameter values. The experiments indicated that model performance plateaued quickly, with negligible improvements beyond the first few epochs. Furthermore, hyperparameter optimization using Optuna (1), which leverages a Tree-structured Parzen Estimator, confirmed that two epochs consistently captured the majority of performance gains. Taken together with the large dataset size, which inherently supports stable generalization, training for two epochs provided reliable results while maintaining a manageable training time.

This study evaluates two reconstruction pipelines: a multiple model pipeline and a single model pipeline. These configurations were investigated to assess the ability of a single model to generalize across varying input signal-to-noise ratios (SNRs). As depicted in Figure 2B, both pipelines use clean ECG leads I, II, and V3 from the same patient record, which are combined with WGN at varying levels (ranging from 0 dB to 40 dB) to serve as the input. The corresponding clean leads V1, V2, V4, V5, and V6 from the same record are used as the target output for reconstruction.

#### 2.2.1 Multiple model pipeline (MMP)

The Multiple Model Pipeline (MMP) is based on the premise that a single model may not be sufficient to accurately reconstruct clean ECG leads from inputs affected by varying levels of noise. To address this, the input signal-to-noise ratio (SNR) range, spanning from 0 dB to 40 dB, is divided into five distinct intervals:

- 0–4 dB (Model 1)
- 5–9 dB (Model 2)
- 10–14 dB (Model 3)
- 15–19 dB (Model 4)
- 20–40 dB (Model 5)

Multiples of the dataset is made with each added noise such that there are 10,000 records for each of the 25 noise levels (0–20 dB, 25 dB, 30 dB, 35 dB and 40 dB). Each model is specifically trained with all the dataset, with all the noise levels within its specified range, to reconstruct clean ECG signals from inputs falling within its assigned SNR range. As a result, the MMP consists of five separate models, each optimized for a particular level of input noise (Figures 2C,D).

#### 2.2.2 Single model pipeline (SMP)

Conversely, the Single Model Pipeline (SMP) operates on the premise that neural networks possess the versatility to learn from and generalize across a broad range of input SNRs. In this approach, a single model is trained using each signal in the dataset with randomly chosen SNR level, spanning from 0 dB to 40 dB in 4 dB intervals. The goal is for the model to accurately reconstruct the output ECG leads regardless of the level of noise present in the input signals. By adopting this approach, the reconstruction framework is simplified considerably, as the SMP employs a single model to handle the full range of noise conditions rather than relying on multiple specialised models. In contrast, the MMP consists of five independent models with identical architectures, each optimised for a specific noise level. Consequently, the MMP requires additional stages for noise classification, model selection, parameter storage, and execution management, thereby increasing pipeline complexity, memory requirements, and computational overhead. The SMP eliminates the need for these auxiliary components by consolidating the reconstruction task into a single generalised model. This design reduces the overall pipeline size by more than fivefold while simplifying deployment and maintenance. The trade-off of this approach is that a single model must learn to generalise across a broader range of signal qualities, rather than benefiting from the specialised optimisation available in the MMP. Nevertheless, the SMP aims to achieve reconstruction performance comparable to that of the more complex multi-model framework while maintaining a substantially leaner system architecture.

### 2.3 System requirements

The models were trained and tested using python on ALICE High Performance Computer at the University of Leicester, UK. Each model was trained with a system containing 24 CPUs, 1 GPU and 35GB memory.

### 2.4 Model analysis

A patient-wise 5-fold cross-validation was conducted for each model across all input noise levels using SET1 dataset. In this procedure, each model was trained five times, with a different 80% subset of the patient records used for training and the remaining 20% reserved for testing in each fold. The final performance metrics were calculated by averaging the results across all five folds. During training, noise was added to each record at random levels within the specific SNR range assigned to the model. For testing, each record in the test set was evaluated across all relevant noise levels corresponding to the model's designated noise group. To prevent data leakage, all signals underwent a standardized denoising process prior to further analysis. A consistent normalization scale was established at the onset of model design and then applied independently to each signal during both training and testing. Furthermore, strict patient-level separation was enforced, ensuring that no data from the same individual appeared simultaneously in both training and testing sets.

#### 2.4.1 Model evaluation on foreign dataset

To further evaluate the model, it was trained on the complete SET1 dataset and tested on the latter record of the 20 patients in SET2. This provided an initial assessment of its performance on a different dataset and under varying heart condition. Additionally, building on the findings of Malpani et al. (17) and Obianom et al. (23) which demonstrated that transfer learning of reconstruction models improves correlation coefficients up to 0.02 over the original generalised models for up to two years, transfer learning will be incorporated in this study. Transfer learning (in this study) is a technique in which a pre-trained model is fine-tuned using a smaller patient-specific dataset to enable the adaptation of a generalised model to a specific patient (12). In this work, the fully trained model (model trained on SET1) will be fine-tuned with the initial record of each patient in SET2 and testing on their latter record. That is, for each patient in SET2, their initial ECG record will be used to retrain the fully trained model, then the fine-tuned model will be tested on the patient's latter ECG record. This approach allows evaluation of patient-specific performance improvement, how this can be achieved with limited patient data, and how the performance compares to the performance of the generic model.

#### 2.4.2 Performance metrics

The performance metrics used to compare these pipelines include the Pearson correlation coefficient and root mean squared error (RMSE). Correlation is usually used to define the degree to which two signals are linearly related. It defines how morphologically similar the reconstructed ECG signal is to the original, indicating how well the waveform features such as *P* waves, QRS complexes, and T waves are preserved for accurate clinical interpretation. Its value ranges from −1 to 1, where 0 means no correlation, −1 means negatively correlated, and 1 means positively correlated. This is mathematically shown in Equation 1.

$$r=\frac{\sum (x_{j}-\hat{x})(y_{j}-\hat{y})}{\sqrt{\sum {\left(x_{j}-\hat{x}\right)}^{2}\sum {\left(y_{j}-\hat{y}\right)}^{2}}}$$ (1)

where:r=correlationcoefficient

j=observationnumberatanygivenpointintime

$$x_{j}=original\;sample\;at\;any\;given\;point\;in\;time$$

$$\hat{x}=mean\;of\;all\;original\;values$$

$$y_{j}=predicted\;sample\;at\;any\;given\;point\;in\;time$$

$$\hat{y}=mean\;of\;all\;predicted\;values$$

RMSE is a metric used to measure the average deviation between the predicted outputs of a model and the actual reference values, focusing on the voltage amplitudes. It measures the average difference between the predicted model and the actual values providing an interpretable measure of the average reconstruction error in the same units as the ECG signal, making it useful for assessing whether deviations are within clinically acceptable limits. A low RMSE indicates that the reconstructed signal closely matches the true electrical potentials of the heart, which is crucial for correctly identifying subtle changes in ECG component waves, while a higher RMSE reflects larger errors and reduced performance and reliability. Unlike correlation, which emphasizes the similarity in waveform shape, RMSE directly captures the accuracy of the reconstructed leads in terms of their amplitude values. The RMSE is calculated by finding the root of the MSE (Equation 2) as shown in Equation 3.

$$MSE=\frac{1}{N}\overset{N}{\underset{\;j=1}{\sum }}(y_{j}-x_{j}{)}^{2}$$ (2)

$$RMSE=\sqrt{MSE}$$ (3)

where:N=totalnumberofobservations

## 3 Results

To understand the performance of a single model in the presence of diverse noise a single model pipeline (SMP) and multiple model pipeline (MMP) were designed with 10,000 records from CODE-15% database (29) (SET1) and five-fold cross-validated across different noise levels. Furthermore, a SET2 was extracted from PTB-XL database (11, 33) and used to test the pipeline for performance on foreign dataset, and the improvements transfer learning can have on the models.

### 3.1 Comparison between MMP and SMP

The results obtained for each model across all leads, in terms of correlation coefficient (r) and RMSE for every patient were averaged. The mean values of these results are presented in Tables 2, 3. The tables show the averaged r and RMSE for each synthesised lead based on the SNR of the input leads. The last two columns show the average across all leads based on the input SNR. In addition, an initial column is added to Table 2 to differentiate results based on the model used to synthesise the leads.

**Table 2:** Average correlation (r) and root mean square error (RMSE) of the reconstruction of the multiple model pipeline (MMP) as compared to the expected output.

Model ⬇	Leads ➔	V1		V2		V4		V5		V6		Average	
	Input SNR ⬇	r	RMSE (mV)	r	RMSE (mV)	r	RMSE (mV)	r	RMSE (mV)	r	RMSE (mV)	r	RMSE (mV)
M1	0dB	0.842	0.150	0.793	0.227	0.853	0.214	0.905	0.180	0.903	0.163	0.859	0.187
	1dB	0.844	0.149	0.798	0.223	0.860	0.207	0.910	0.176	0.907	0.160	0.864	0.183
	2dB	0.845	0.147	0.801	0.221	0.865	0.203	0.912	0.174	0.909	0.158	0.867	0.181
	3dB	0.846	0.147	0.803	0.220	0.869	0.199	0.915	0.173	0.911	0.157	0.869	0.179
	4dB	0.846	0.147	0.805	0.220	0.870	0.198	0.916	0.172	0.912	0.157	0.870	0.179
M2	5dB	0.845	0.147	0.817	0.215	0.868	0.202	0.918	0.172	0.915	0.157	0.872	0.178
	6dB	0.847	0.146	0.819	0.213	0.872	0.198	0.920	0.169	0.916	0.155	0.875	0.176
	7dB	0.847	0.145	0.821	0.212	0.875	0.195	0.921	0.167	0.917	0.153	0.876	0.175
	8dB	0.847	0.145	0.822	0.211	0.877	0.193	0.922	0.166	0.918	0.153	0.877	0.174
	9dB	0.846	0.145	0.822	0.211	0.879	0.192	0.923	0.165	0.918	0.152	0.877	0.173
M3	10dB	0.849	0.145	0.823	0.212	0.879	0.194	0.922	0.167	0.917	0.154	0.878	0.175
	11dB	0.850	0.145	0.824	0.211	0.880	0.193	0.923	0.166	0.917	0.154	0.879	0.174
	12dB	0.850	0.144	0.825	0.211	0.880	0.193	0.923	0.166	0.918	0.153	0.879	0.173
	13dB	0.851	0.144	0.826	0.210	0.881	0.192	0.924	0.165	0.918	0.153	0.880	0.173
	14dB	0.851	0.144	0.826	0.210	0.881	0.191	0.924	0.165	0.919	0.153	0.880	0.173
M4	15dB	0.852	0.143	0.826	0.208	0.880	0.192	0.923	0.164	0.918	0.151	0.880	0.172
	16dB	0.853	0.143	0.827	0.207	0.880	0.192	0.924	0.164	0.919	0.151	0.880	0.171
	17dB	0.853	0.143	0.827	0.207	0.880	0.191	0.924	0.164	0.919	0.151	0.881	0.171
	18dB	0.853	0.143	0.827	0.207	0.880	0.191	0.924	0.164	0.919	0.151	0.881	0.171
	19dB	0.853	0.143	0.828	0.207	0.880	0.191	0.924	0.164	0.919	0.151	0.881	0.171
M5	20dB	0.858	0.142	0.830	0.207	0.886	0.187	0.925	0.162	0.919	0.151	0.884	0.170
	25dB	0.859	0.142	0.831	0.207	0.887	0.187	0.926	0.162	0.920	0.150	0.884	0.169
	30dB	0.859	0.142	0.831	0.207	0.887	0.186	0.926	0.162	0.920	0.150	0.885	0.169
	35dB	0.859	0.142	0.831	0.207	0.887	0.186	0.926	0.162	0.920	0.150	0.885	0.169
	40dB	0.859	0.142	0.831	0.207	0.887	0.186	0.926	0.162	0.920	0.150	0.885	0.169

**Table 3:** Average correlation (r) and root mean square error (RMSE) of the reconstruction of the single model pipeline (SMP) as compared to the expected output.

Leads ➔	V1		V2		V4		V5		V6		Average	
Input SNR ⬇	r	RMSE (mV)	r	RMSE (mV)	r	RMSE (mV)	r	RMSE (mV)	r	RMSE (mV)	R	RMSE (mV)
0dB	0.832	0.158	0.792	0.232	0.851	0.217	0.900	0.187	0.900	0.167	0.855	0.192
1dB	0.839	0.154	0.799	0.227	0.858	0.211	0.905	0.182	0.904	0.163	0.861	0.187
2dB	0.843	0.151	0.805	0.223	0.863	0.207	0.909	0.178	0.907	0.160	0.866	0.184
3dB	0.847	0.149	0.810	0.220	0.867	0.203	0.911	0.175	0.910	0.158	0.869	0.181
4dB	0.850	0.147	0.814	0.217	0.870	0.201	0.914	0.173	0.911	0.157	0.872	0.179
5dB	0.851	0.146	0.818	0.215	0.872	0.199	0.916	0.171	0.913	0.156	0.874	0.177
6dB	0.852	0.145	0.821	0.213	0.873	0.198	0.917	0.170	0.914	0.155	0.875	0.176
7dB	0.853	0.145	0.823	0.211	0.875	0.196	0.918	0.169	0.915	0.154	0.877	0.175
8dB	0.853	0.144	0.825	0.210	0.876	0.195	0.920	0.168	0.916	0.153	0.878	0.174
9dB	0.853	0.144	0.826	0.209	0.877	0.194	0.920	0.167	0.917	0.153	0.879	0.173
10dB	0.853	0.144	0.827	0.208	0.878	0.193	0.921	0.166	0.917	0.152	0.879	0.173
11dB	0.853	0.144	0.828	0.207	0.879	0.193	0.922	0.166	0.918	0.152	0.880	0.172
12dB	0.852	0.144	0.829	0.207	0.879	0.192	0.922	0.165	0.918	0.152	0.880	0.172
13dB	0.852	0.145	0.829	0.207	0.879	0.192	0.922	0.165	0.918	0.152	0.880	0.172
14dB	0.851	0.145	0.829	0.206	0.880	0.191	0.922	0.165	0.918	0.152	0.880	0.172
15dB	0.851	0.145	0.830	0.206	0.880	0.191	0.923	0.165	0.919	0.152	0.880	0.172
16dB	0.851	0.145	0.830	0.206	0.880	0.191	0.923	0.165	0.919	0.152	0.880	0.172
17dB	0.851	0.145	0.830	0.206	0.880	0.191	0.923	0.165	0.919	0.152	0.880	0.172
18dB	0.850	0.145	0.830	0.206	0.880	0.191	0.923	0.165	0.919	0.152	0.880	0.172
19dB	0.850	0.145	0.831	0.206	0.880	0.191	0.923	0.165	0.919	0.152	0.880	0.172
20dB	0.850	0.146	0.831	0.206	0.880	0.191	0.923	0.165	0.919	0.152	0.881	0.172
25dB	0.850	0.146	0.831	0.206	0.880	0.191	0.923	0.165	0.919	0.152	0.881	0.172
30dB	0.850	0.146	0.831	0.206	0.880	0.191	0.923	0.165	0.919	0.152	0.881	0.172
35dB	0.850	0.146	0.831	0.206	0.880	0.191	0.923	0.165	0.919	0.152	0.881	0.172
40dB	0.850	0.146	0.831	0.206	0.880	0.191	0.923	0.165	0.919	0.152	0.881	0.172

Both the MMP and SMP exhibited similar trends in terms of correlation coefficient (r) and RMSE. As the input SNR increased, the average r increased and the average RMSE decreased. The range of the average r for both pipelines was also 0.026. The range of the average RMSE for the MMP and SMP was 0.018 and 0.02 respectively. The ranges show very little difference in performance at lower input SNR in comparison to higher input SNR.

Finally, a paired t-test was performed on the average correlation of both pipelines to aid the identification the promising reconstruction pipeline for further development. Since reconstruction without denoising is a novel approach, it is worth deciding at the early stages (which is the focus of this paper) the best direction for the development of this reconstruction technique in the future. The *p*-value in this work serves to indicate difference between pipelines rather than establishing broader claims of clinical or practical significance. The paired t-test showed no significant difference for 0.05 threshold on the *p*-value. This suggests no difference in the two pipelines on capturing the pattern of the synthesised leads. However, the average RMSE between both pipelines was significantly different. This indicates the accuracies of both models are different, that is, MMP is more accurate than SMP.

### 3.2 Testing SMP on foreign dataset

Owing to the similarity between the results of the MMP and SMP, and the simplicity of the SMP, the foreign dataset was only applied to the SMP. The SMP was trained on the full SET1. The fully trained model was tested on SET2, in its generic form and when fine-tuned using transfer learning. The results of the generic (Gen) and fine-tuned (Tran) models across all leads, in terms of correlation coefficient (r) and RMSE for all patients was averaged. The mean values of these results are presented in Table 4. The table is organised to show the results for normal and MI patients across input noise levels 0 dB to 40 dB in steps of 4 dB. 17 dB to 39 dB is not shown because the results are very similar and there is little difference in the morphology of the dataset.

**Table 4:** Average correlation (r) and root mean square error (RMSE) of the reconstruction of the single model pipeline (SMP) trained on CODE-15% database and tested on PTB-XL database. Gen represents results gotten when the generic model was used. Tran represents results gotten when the generic model was fine-tuned (transfer learning performed) for each patient.

Condition	Input SNR ⬇	Leads ➔	V1		V2		V4		V5		V6		Average	
		Model Type ⬇	r	RMSE (mV)	r	RMSE (mV)	r	RMSE (mV)	r	RMSE (mV)	r	RMSE (mV)	r	RMSE (mV)
Normal	0dB	Gen	0.655	0.099	0.717	0.177	0.932	0.125	0.938	0.147	0.828	0.141	**0** **.** **814**	**0**.**138**
		Tran	0.841	0.074	0.822	0.124	0.928	0.100	0.935	0.087	0.830	0.089	**0**.**871**	**0**.**095**
	4dB	Gen	0.712	0.095	0.752	0.171	0.950	0.125	0.952	0.155	0.840	0.146	**0**.**841**	**0**.**138**
		Tran	0.886	0.065	0.868	0.107	0.948	0.089	0.949	0.081	0.843	0.083	**0**.**899**	**0**.**085**
	8dB	Gen	0.761	0.087	0.785	0.165	0.960	0.108	0.963	0.139	0.847	0.137	**0**.**863**	**0**.**127**
		Tran	0.913	0.055	0.897	0.090	0.969	0.064	0.968	0.057	0.857	0.071	**0**.**921**	**0**.**067**
	12dB	Gen	0.764	0.081	0.786	0.159	0.962	0.090	0.970	0.119	0.851	0.126	**0**.**867**	**0**.**115**
		Tran	0.930	0.045	0.907	0.079	0.980	0.047	0.978	0.041	0.863	0.064	**0**.**932**	**0**.**055**
	16dB	Gen	0.761	0.081	0.788	0.155	0.962	0.090	0.971	0.118	0.852	0.126	**0**.**867**	**0**.**114**
		Tran	0.935	0.043	0.907	0.075	0.983	0.045	0.981	0.038	0.866	0.062	**0**.**934**	**0**.**053**
	40dB	Gen	0.771	0.079	0.798	0.151	0.963	0.086	0.972	0.113	0.852	0.124	**0**.**871**	**0**.**111**
		Tran	0.938	0.042	0.914	0.070	0.984	0.044	0.982	0.037	0.867	0.061	**0**.**937**	**0**.**051**
Infarction	0dB	Gen	0.579	0.144	0.500	0.150	0.691	0.120	0.677	0.112	0.654	0.083	**0**.**620**	**0**.**122**
		Tran	0.761	0.117	0.764	0.109	0.760	0.103	0.748	0.101	0.720	0.082	**0**.**751**	**0**.**102**
	4dB	Gen	0.588	0.143	0.501	0.149	0.686	0.120	0.675	0.116	0.682	0.086	**0**.**627**	**0**.**123**
		Tran	0.809	0.105	0.795	0.100	0.805	0.094	0.782	0.097	0.757	0.079	**0**.**790**	**0**.**095**
	8dB	Gen	0.597	0.143	0.520	0.146	0.673	0.121	0.612	0.119	0.647	0.086	**0**.**610**	**0**.**123**
		Tran	0.835	0.097	0.835	0.087	0.830	0.085	0.797	0.086	0.786	0.064	**0**.**817**	**0**.**084**
	12dB	Gen	0.618	0.141	0.528	0.146	0.668	0.118	0.565	0.116	0.597	0.082	**0**.**595**	**0**.**121**
		Tran	0.847	0.093	0.831	0.084	0.842	0.076	0.794	0.079	0.791	0.056	**0**.**821**	**0**.**078**
	16dB	Gen	0.614	0.141	0.529	0.145	0.669	0.115	0.554	0.114	0.584	0.080	**0**.**590**	**0**.**119**
		Tran	0.851	0.092	0.827	0.083	0.841	0.075	0.797	0.076	0.801	0.053	**0**.**823**	**0**.**076**
	40dB	Gen	0.613	0.141	0.533	0.145	0.668	0.114	0.554	0.112	0.584	0.079	**0**.**590**	**0**.**118**
		Tran	0.853	0.091	0.829	0.082	0.842	0.074	0.794	0.076	0.798	0.052	**0**.**823**	**0**.**075**

Bold values represent the mean reconstruction performance across all patient conditions at a given noise level, providing an overall comparison of the Generic (Gen) and Transfer Learning (Tran) models.

The generic model consistently underperformed compared to the fine-tuned model, showing lower correlation and higher RMSE in all cases. This gap was more pronounced for MI patients than for normal ECGs. Despite fine-tuning, the models still performed worse on MI patients compared to those with normal ECGs. This discrepancy may stem from limitations in the training data, and further analysis with more diverse datasets is needed to fully understand the underlying causes. Overall, the fine-tuned model delivered superior accuracy, highlighting the importance of patient-specific adaptation for reliable reconstruction, especially in complex cardiac conditions.

## 4 Discussion

### 4.1 Comparison between MMP and SMP

**Figure 3:**
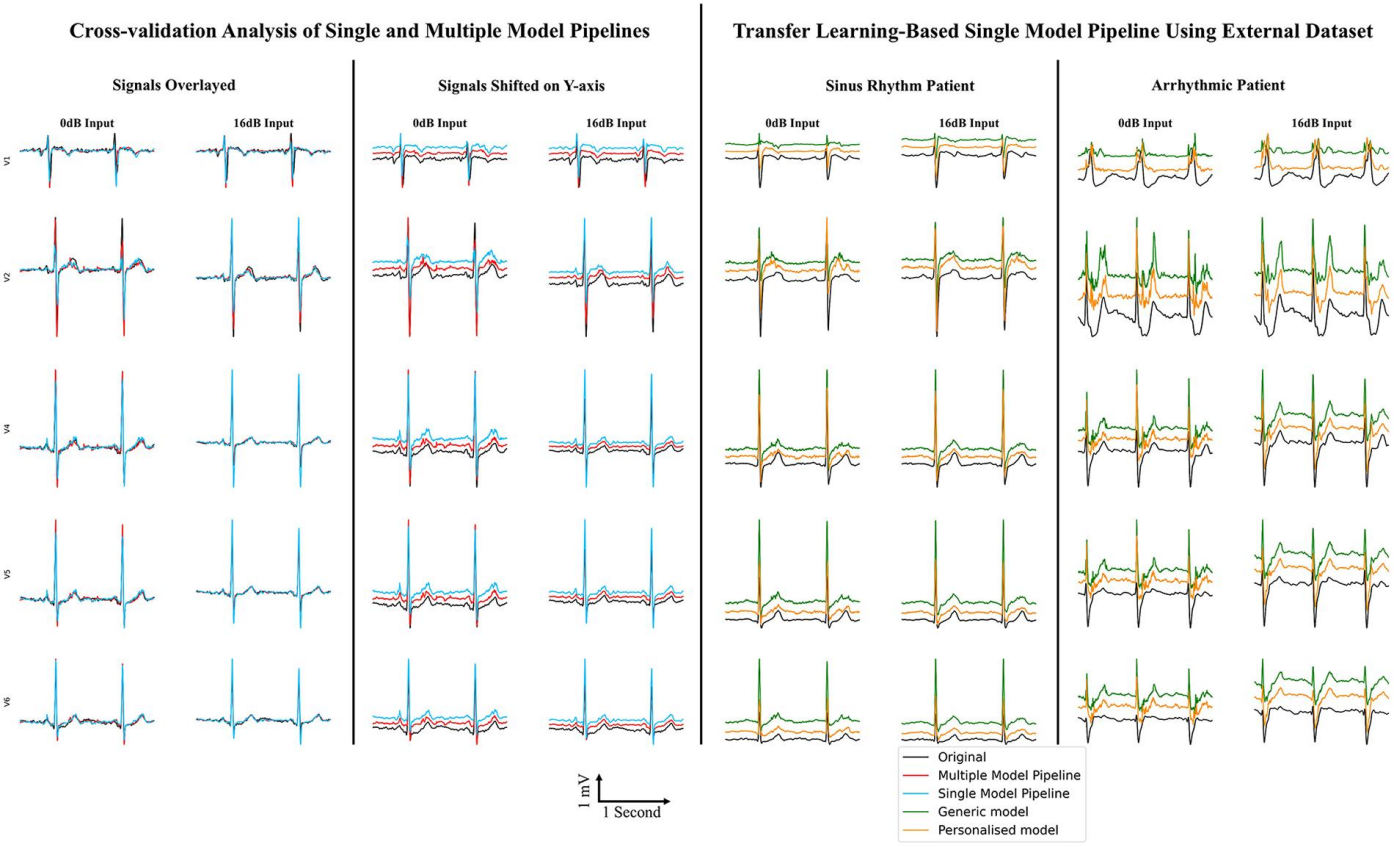
Sample reconstruction of a randomly selected test record under input SNR conditions of 0 dB and 15 dB, where the black trace denotes the baseline denoised ECG signal prior to noise injection, the red trace denotes the reconstruction produced by the multiple model pipeline (MMP), the blue trace denotes the single model pipeline (SMP), the green trace denotes the SMP trained on an external dataset without adaptation, and the yellow trace denotes the personalised SMP following transfer learning on the external dataset; the left panel presents cross-validation comparisons between MMP and SMP. The far-left sub-panel showing overlaid original and reconstructed signals where increased overlap indicates higher reconstruction similarity, and the sub-panel next to it repeats the same signals with vertical offsets for clearer visualisation. The right panel illustrates the effect of transfer learning on SMP performance, where its left sub-panel corresponds to a rhythmic patient and the right sub-panel corresponds to an arrhythmic patient, comparing the generic SMP with its personalised variant. The reconstructed signals are vertically offset (0.2 mV and 0.4 mV) to improve morphological clarity; the vertical axis is expressed in millivolts (mV) and the horizontal axis in seconds.

**Figure 4:**
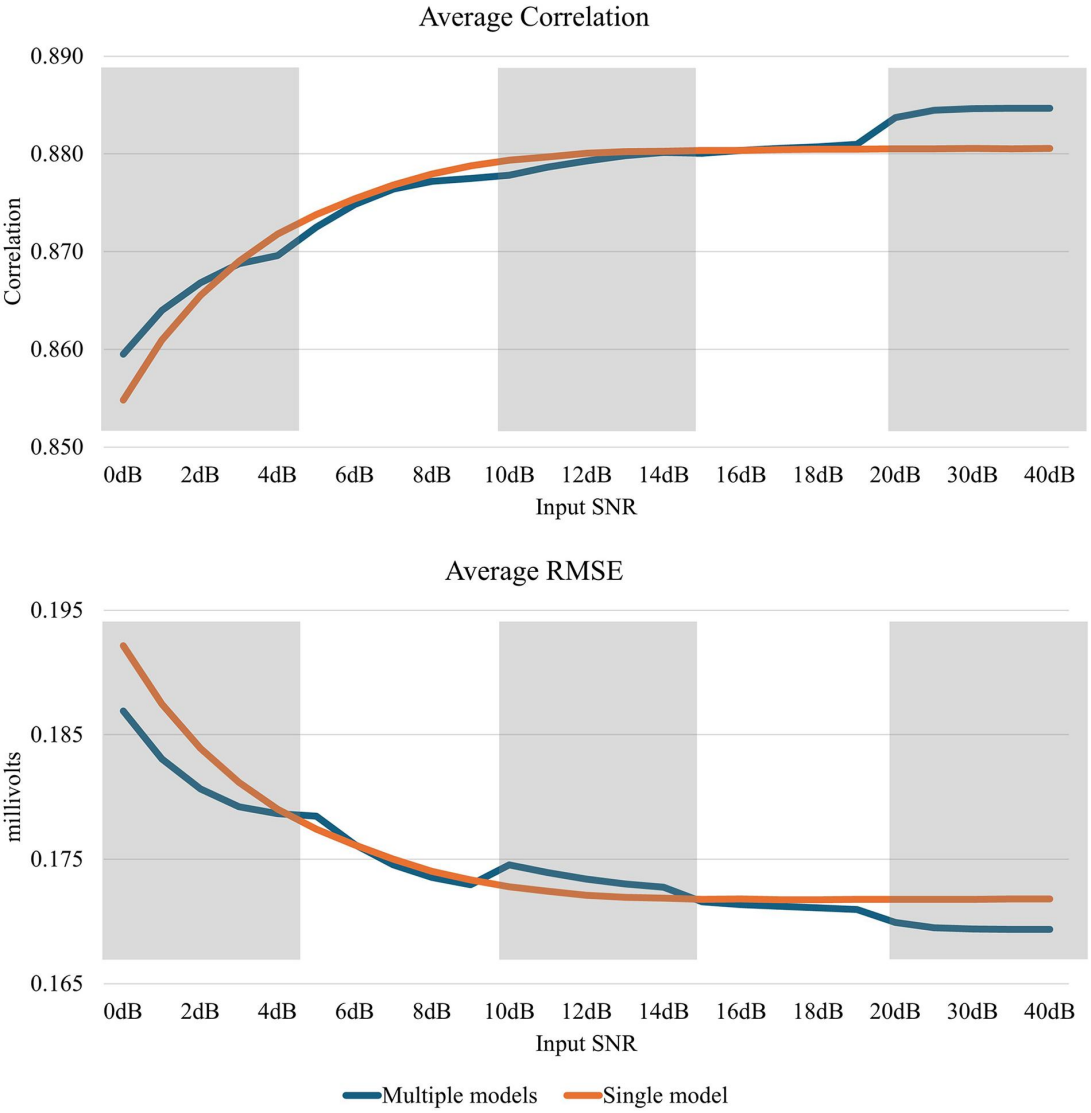
Figure showing the trend of the average correlation and average RMSE across all leads of the single model pipeline (SMP) and multiple model pipeline (MMP) with relation to the input SNR. The SMP is denoted in orange, while the MMP is denoted in blue. The white or grey vertical shade denotes the different input SNR segments specialised on by the different MMP models.

Based on the results of the t-test, the RMSE between the two pipelines on a lead-to-lead basis is statistically different, whereas the correlation coefficients are not. This signifies that the reconstructed leads of both the SMP and MMP have similar morphology, but the predicted electrical potentials may differ. However, the magnitude of the RMSE difference is relatively small and would typically go unnoticed, as illustrated in Figure 3. Given that the amplitude of a standard ECG signal is approximately 1 mV, an isoline difference of 0.003 mV (which corresponds to the difference between the average RMSE values of the two pipelines at an input SNR of 40 dB) would appear negligible. In clinical practice, greater emphasis is placed on the morphology, rhythm patterns, and relative timing of the ECG rather than minor amplitude variations (2, 27), suggesting that waveform shape and temporal consistency are more diagnostically relevant than small differences in signal magnitude. Correlation therefore serves as a stronger proxy for clinically relevant similarity, as it reflects alignment in morphology, rhythm, and timing, whereas RMSE is more sensitive to absolute amplitude deviations between signals. This therefore, suggests that if a clinician were to independently assess the reconstructions from both pipelines, there is a good chance that they would be unable to distinguish between them. However, this claim still requires clinical assessment for validation. It is also worth noting (as seen in Figure 4) that SMP performed worse than MMP at both lower and higher input SNR levels. This outcome is likely attributable to the differing architecture and training requirements of MMP models across varying SNR conditions. These findings underscore the potential relevance of SMP, as it allows resources to be concentrated on developing a single, more versatile model capable of reconstructing signals across a wide range of noise levels, rather than having to address the distinct architecture and training requirements of multiple models in the MMP framework.

This paper builds upon the preliminary study conducted by Obianom et al. (22), which investigated the performance of LSTM models relative to linear regression across different noise levels. A notable similarity can be observed between Figure 4 and the corresponding figures in that study, particularly in terms of average correlation and RMSE values. However, this work advances the previous findings by proposing that a single model can achieve comparable performance across varying noise levels, a conclusion supported by the results presented. Furthermore, when compared to prior ECG reconstruction studies, the relevance of this approach becomes even more apparent. As reported in Obianom et al. (24), most non-linear ECG reconstruction models (14, 34, 35) achieve average correlation values between 0.85 and 0.9, aligning closely with the performance observed for the pipeline proposed in this paper (Table 1). This reinforces the idea that a model trained across a range of noise levels can perform to the same standard as models specifically trained on clean, denoised ECG signals.

### 4.2 Comparison between SMP and traditional methods

To further evaluate the efficacy of the SMP, its performance was compared against several traditional ECG reconstruction approaches previously investigated using the same SET1 dataset. These approaches represent a range of reconstruction paradigms commonly reported in the literature and include the linear transformation method (16), the wave masking pipeline (25), a feed forward neural network based approach (4), and a LSTM architecture derived from (8) and further enhanced for this study. The comparative results presented in Table 5 include an extract of the previously reported performances as shown presented by Obianom et al. (25) alongside the results obtained by the SMP at an input SNR of 40 dB.

**Table 5:** Lead-wise reconstruction performance (average correlation) using five-fold cross-validation, comparing the proposed SMP against classical and machine learning-based ECG reconstruction methods under identical evaluation conditions.

Model	Linear transformation Maheshwari et al. (16)	Wave masked linear transformation Obianom et al. (25)	Feed forward network Atoui et al. (4)	SMP (Proposed)
V1	0.896	0.843	0.867	0.850
V2	0.810	0.825	0.847	0.831
V4	0.827	0.895	0.903	0.880
V5	0.899	0.918	0.928	0.923
V6	0.913	0.918	0.928	0.919
Average	0.869	0.880	0.895	0.881

As summarised in Table 1, most previous ECG reconstruction studies rely on cascaded denoising and preprocessing pipelines prior to reconstruction. Similarly, the comparative models evaluated in the referenced study were trained using conventionally denoised input signals. In contrast, the SMP proposed in this work was intentionally trained using noisy input data across varying noise conditions without requiring a dedicated denoising stage before reconstruction. This design enables the model to learn reconstruction features directly under noisy conditions rather than depending on heavily constrained input representations.

The results demonstrate that the SMP achieved comparable reconstruction performance to the traditional approaches evaluated, as shown in Table 5. This indicates that similar levels of reconstruction fidelity can be achieved without relying on an explicit denoising stage within the preprocessing pipeline. A likely explanation is that exposure to noisy inputs during training improves the model's robustness to signal variability and acquisition artefacts, thereby reducing susceptibility to unexpected perturbations during inference. Rather than depending on preprocessing to standardise the signal prior to reconstruction, the SMP learns to directly map noisy inputs to reconstructed outputs, effectively internalising noise variability within the learning process itself. This contributes to stable generalisation across varying signal conditions. Furthermore, unlike traditional pipelines where performance is coupled to the effectiveness of the denoising stage, the SMP operates as a simplified end-to-end framework with reduced preprocessing dependency. This reduces pipeline complexity, particularly in resource-constrained environments where computational efficiency and minimal preprocessing overhead are critical considerations.

Although the proposed SMP eliminates the explicit denoising stage present in conventional reconstruction pipelines, a direct quantitative comparison of computational speed was not performed. This is because the models evaluated in this study were trained and executed under different computational configurations within a high-performance computing (HPC) environment, where execution time was strongly influenced by queue allocation, batch scheduling, model complexity, and distributed workload management rather than solely by reconstruction architecture. Furthermore, several traditional methods were reproduced from prior studies and adapted for execution on the HPC system. To manage computational constraints and reduce queue delays, training was restructured into independently executed batches, effectively operating on a one-epoch-per-batch style workflow. While this improved training feasibility, it also made runtime heavily dependent on HPC scheduling and job management rather than model efficiency alone. As a result, direct and fair timing comparisons between pipelines were not possible. Nevertheless, removing the denoising stage reduces preprocessing overhead and simplifies the overall reconstruction pipeline, which is expected to improve computational efficiency during deployment.

### 4.3 Evaluation of SMP generalisability and transfer learning insight

**Figure 5:**
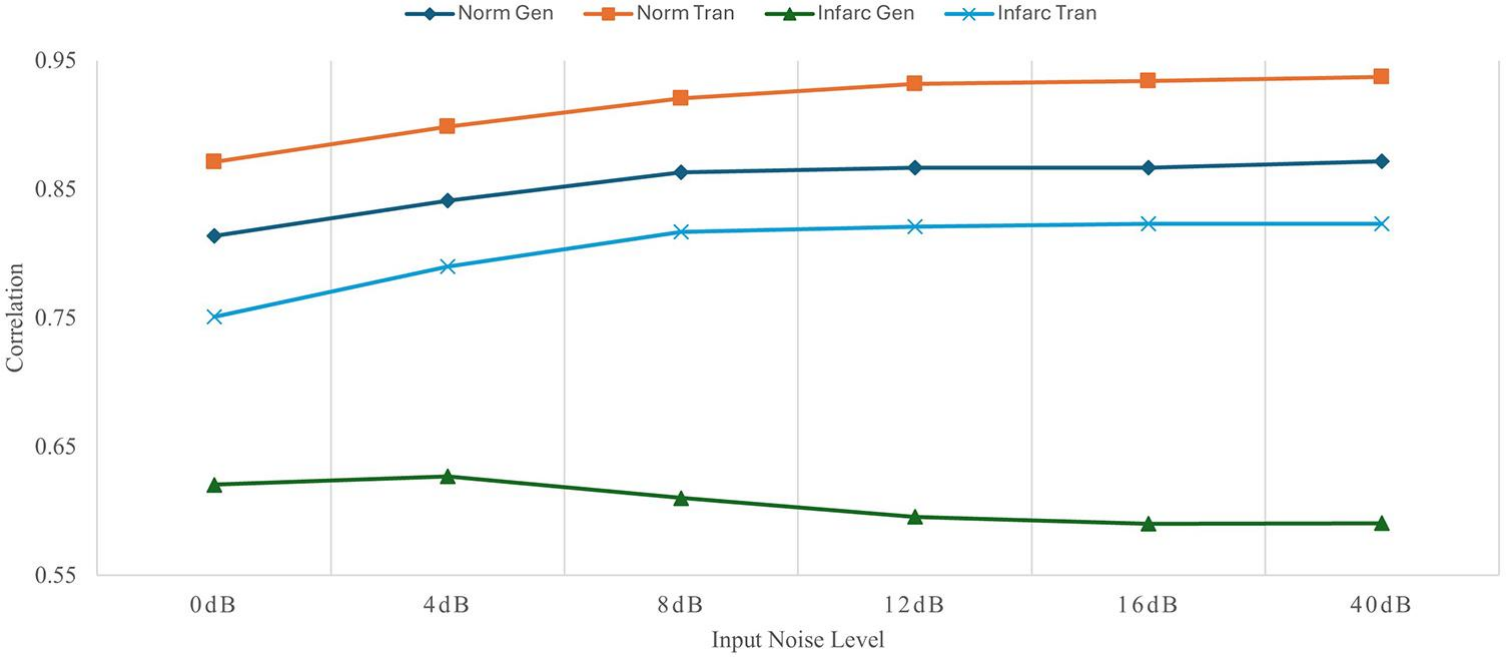
Trend of the average correlation across all leads for the fully trained single model pipeline (SMP) with relation to the input SNR. The SMP is evaluated on 10 normal and 10 myocardial infarction patient records from the PTB-XL database. Tests were conducted on the model in its generic form and in its fine-tuned (through transfer learning) form. In the legend, ‘Norm’ indicates evaluation on normal records, ‘Infarc’ denotes evaluation on myocardial infarction records, ‘Gen’ denotes evaluation with generic model, and ‘Tran’ denotes evaluation with fine-tuned model.

The SMP was trained on the complete SET1 dataset and evaluated on SET2, a distinct dataset. Testing on normal ECG records produced results closely aligned with the model's 5-fold cross-validation performance (as seen in Figures 3–5). Specifically, the average correlation coefficients ranged from 0.814 to 0.871 across noise levels incrementally increasing from 0 dB to 20 dB. Although slightly lower than the validation results, these values remain consistent with the expected range.

In contrast, performance on MI records was substantially poorer. Correlation values fluctuated between 0.59 and 0.62, highlighting a significant drop in accuracy (Figure 5). This discrepancy can be attributed to the composition of the training dataset, which primarily contained normal ECG signals. Consequently, the model was optimized to reconstruct normal ECG patterns from noise-contaminated normal ECG and lacked the capacity to accurately reconstruct abnormal signals. This limitation is inherent to models trained without exposure to pathological variations. This finding underscores the importance of incorporating representative pathological data during training or leveraging adaptive fine-tuning strategies to enhance generalization across diverse clinical conditions.

When the SMP was retrained using a small subset of patient-specific data, its performance improved significantly. As illustrated in Figure 5, correlation values for both normal and MI ECG reconstructions increased substantially, ranging from 0.871 to 0.937 for normal ECG and 0.751–0.823 for MI ECG. These findings highlight two critical insights. First, they reinforce prior evidence that patient-specific models consistently outperform generic models, as individual cardiac dynamics vary significantly across patients. A model tailored to these unique characteristics will invariably yield superior reconstruction accuracy (17, 23). Second, the results point to the importance of training data composition. Notably, the initial model did not require exposure to abnormal rhythms during its primary training phase to achieve significant improvements upon retraining. Furthermore, only a minimal amount of patient data (potentially as little as a 10-second ECG recording at 500 Hz) was sufficient to adapt the model effectively. This opens promising avenues for clinical application, where a broadly trained generic reconstruction model can be deployed and subsequently fine-tuned for individual patients, leveraging publicly available datasets regardless of geographic or pathological variability. Nevertheless, this requires more research as the testing data here is 10 ‘normal’ and 10 ‘MI’ patient records and is not enough to make a conclusive statement.

**Figure 6:**
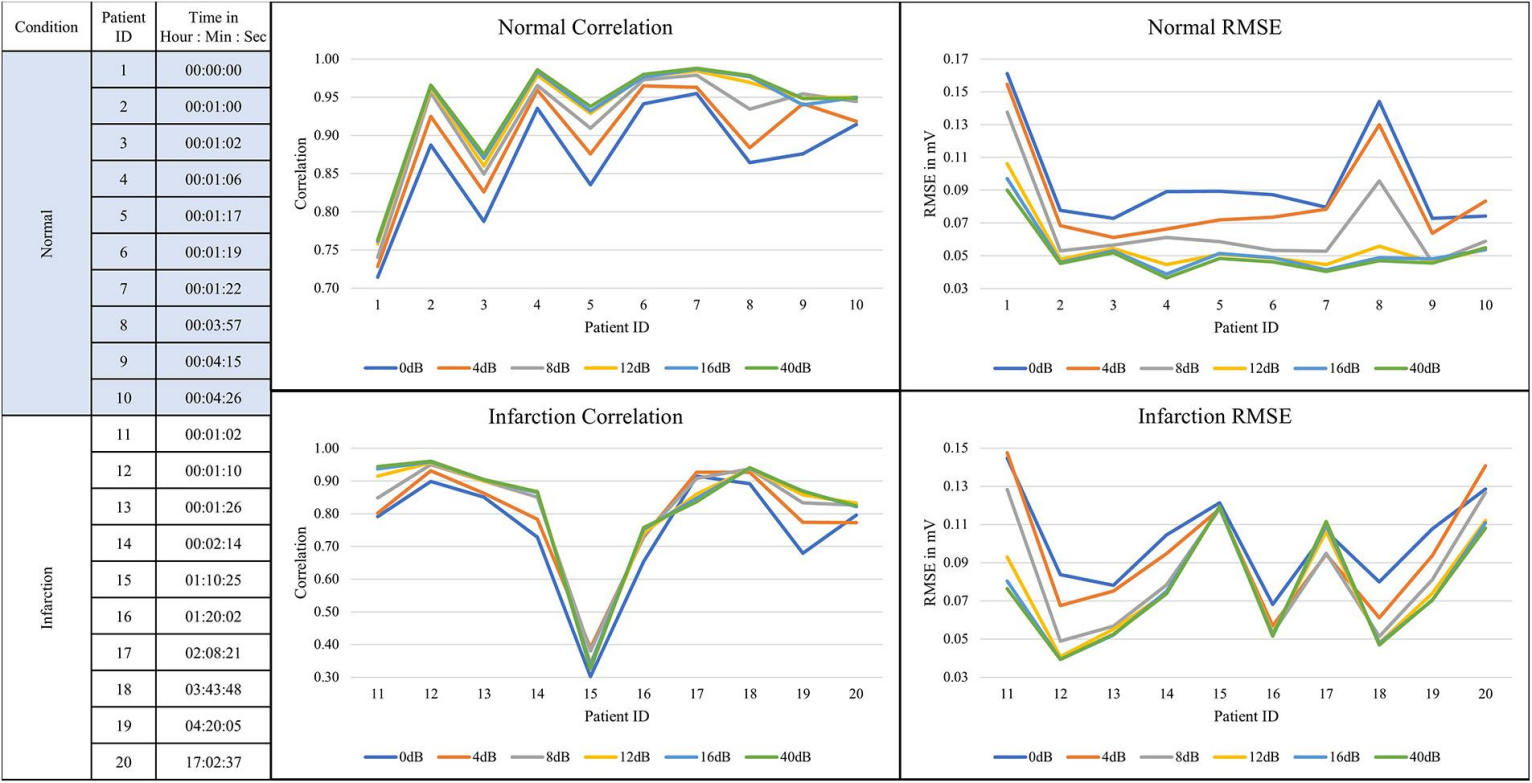
Trend of the average correlation and average RMSE across all leads for each patient after transfer learning with relation to the input SNR. To the left, a table of the time interval between the initial signal used to fine-tune the model and final signal used to test the model. “Normal” represents patients with sinus rhythm while “Infarction” represents patients with myocardial infarction.

Individual patients also showed varying levels of performance irrespective of the time intervals between their recordings. As shown in Figure 6, each patient has a unique time interval between their initial and final recordings, yet there remains substantial variability in reconstruction performance across patients, particularly within the “Infarction” cohort. This suggests that factors beyond time interval contributes to reconstruction variability, including inter-patient physiological differences and signal characteristics. However, a gradual upward performance trend can be observed among the “Normal” patients as the time interval increases, despite the intervals spanning less than 5 min. A similar trend was previously reported in Obianom et al. (23) where fine-tuned (personalised) models evaluated on 50 “Normal” patients had an upward trend over a period extending up to 2 years. A possible explanation for the observed trend is that the patients represented across the different time intervals are not identical, meaning that the apparent temporal relationship may instead reflect inter-patient variability rather than a true time-dependent effect. Consequently, the observed trend may be influenced by differences in individual physiological characteristics and reconstruction difficulty across patients. Therefore, to confidently verify whether reconstruction performance systematically changes with time interval, a longitudinal study using the same patient cohort assessed at predefined intervals over an extended period would be required. Regarding the “Infarction” cohort, reconstruction performance remained relatively consistent across time intervals, except for outlier patient 15. This observation further supports the claim that transfer learning improves model adaptability and that personalised models remain viable for a period following adaptation (17, 23). The relatively stable performance in the infarction group may also indicate that persistent pathological ECG characteristics provide more consistent features for the model to learn and retain over time.

### 4.4 Significance in telemedicine

The potential applications of this technology are substantial, particularly in the field of telemedicine. Consistent with previous studies, the findings support the feasibility of synthesizing full ECG signals from a reduced set of leads. This significantly decreases the need for the traditional ten-electrode setup, as the method proposed in this paper requires only four electrodes. Reducing the number of electrodes not only minimizes the likelihood of electrode misplacement (since fewer electrodes inherently mean fewer placement errors) but also contributes to lowering both the cost of equipment and the time required for setup. Consequently, this approach enhances efficiency and accessibility in clinical and remote monitoring environments.

Secondly, this technology not only reduces the number of electrodes required for ECG recording but also eliminates the need for a separate denoising step. Depending on the algorithm employed, denoising can be a computationally intensive process. By removing this pre-processing requirement, the overall complexity of the reconstruction software is significantly reduced. This simplification is particularly valuable in embedded system designs, such as wearable devices, where every kilobyte of storage is critical. Eliminating the need for dedicated denoising software frees up memory and processing capacity, allowing for the inclusion of additional essential features within the device.

From a storage and efficiency perspective, the SMP has demonstrated itself to be a reliable and practical alternative to the MMP. Each model in the MMP occupies approximately 20MB, resulting in a total storage requirement exceeding 100MB for all five models. In contrast, the SMP requires only a single 20MB model, reducing the storage demand for the reconstruction task by more than a factor of five. Moreover, the MMP would typically require an additional classification model or algorithm to determine the noise level of the input ECG signal as a pre-processing step, further increasing the overall storage and computational requirements. Even in cloud-based implementations, where processing occurs remotely rather than on the recording device, both storage and computational resources incur costs. Therefore, minimizing the number of models and processing steps not only enhances efficiency but also improves cost-effectiveness and responsiveness of the system.

Additionally, most of the computational effort lies in the training phase, while the inference time is extremely small, measured in nanoseconds, and thus virtually imperceptible to the user. It is important to note, however, that the model requires a 100-sample input window (equivalent to 0.2 s) to predict the next sample. In practical terms, this means that in a real-time monitoring scenario, the model will not produce output for the first 0.2 s, after which predictions are generated without additional temporal lag. In most clinical contexts, this is inconsequential, as the average adult heart rate ranges between one and four beats per second. Consequently, during resting conditions with lower heart rates, this corresponds to less than a quarter of a single heartbeat, and even during elevated heart rates it translates to not more than a single heartbeat. Such a minor detail is unlikely to compromise the clinical utility of the system.

### 4.5 Limitations and future work

However, it is important to acknowledge the limitations of this study. The models were trained and evaluated using only white Gaussian noise (WGN) as the source of signal distortion. While WGN serves as a general approximation of the types of noise that can affect ECG signals and provides a controlled baseline for proof-of-concept evaluation, it does not fully capture the complexity of real-world noise sources such as power line interference, baseline wander, motion artefacts, and electromyographic interference. As such, further investigation is needed, and will be performed, to assess the model's robustness and effectiveness when confronted with these additional noise sources. Additionally, the current study focused exclusively on ECGs categorized as ‘normal’ and utilized the CODE-15% dataset, which comprises ECG recordings from patients in Brazil. This approach demonstrated limited generalisability, as the generic models trained on this dataset performed significantly poorer when evaluated on MI records (SET2). This contrasts with the performance reported in the preliminary study by Obianom et al. (22), conducted using the PTB database (6, 11), where comparable reconstruction performance was achieved when training on both normal and MI records.

The findings from the preliminary study, together with the reduced performance observed in the present work, highlight the importance of training reconstruction models on datasets encompassing a wider range of cardiac conditions. Such diversity is likely necessary to improve model robustness and ensure reliable performance in the presence of pathological waveform variations encountered in clinical settings. Expanding the training data to include a broader spectrum of cardiac abnormalities may therefore enhance the adaptability and generalisation capability of fine-tuned models across diverse pathologies. Therefore, future research should systematically investigate the model's adaptability under varying physiological and acquisition conditions, including different cardiac abnormalities and input noise profiles. These efforts would help establish the practical limitations of the SMP model while informing strategies for scalable and clinically reliable deployment.

Finally, the results show the remarkable capability of machine learning algorithms in the context of ECG reconstruction. They demonstrate that, when trained with an appropriate dataset and under the right conditions, a single model can effectively perform a task traditionally handled by multiple models. Notably, there was no significant difference in the correlation of the synthesised leads between the Single Model Pipeline (SMP) and the Multiple Model Pipeline (MMP), reinforcing the efficacy of the SMP approach. Although this study focused specifically on WGN as the source of signal distortion, the findings highlight the potential of deep learning models to close the gap between noisy and clean ECG signals, suggesting a future in which denoising may no longer be a prerequisite for accurate reconstruction.

## Data Availability

The original contributions presented in the study are included in the article/Supplementary Material, further inquiries can be directed to the corresponding authors.
